# SIL-TAL1融合基因阳性急性T淋巴细胞白血病19例临床分析

**DOI:** 10.3760/cma.j.issn.0253-2727.2023.02.008

**Published:** 2023-02

**Authors:** 丽君 王, 玙 陈, 萌 向, 小飞 杨, 苏宁 陈

**Affiliations:** 苏州大学第一附属医院，江苏省血液研究所，国家血液系统疾病临床医学研究中心，苏州 215006 The First Affiliated Hospital of Soochow University, Jiangsu Institute of Hematology, National Clinical Medical Research Center for Hematological Diseases, Suzhou 215006, China

**Keywords:** 白血病，T淋巴细胞，急性, SIL-TAL1融合基因, 造血干细胞移植, 预后, Leukemia, T lymphocytic, acute, SIL-TAL1 fusion gene, Hematopoietic stem cell transplantation, Prognosis

## Abstract

**目的:**

分析伴有SIL-TAL1融合基因急性T淋巴细胞白血病（T-ALL）患者的临床、分子生物学和预后特征，以提高对该疾病的认识。

**方法:**

回顾性分析2014年1月至2022年2月苏州大学附属第一医院收治的19例SIL-TAL1融合基因阳性T-ALL患者临床资料，并与同期收治的196例SIL-TAL1融合基因阴性T-ALL患者比较。

**结果:**

19例SIL-TAL1阳性T-ALL患者中，男16例（84.2％），女3例（15.8％），诊断时中位年龄15（7～41）岁；与SIL-TAL1阴性患者相比，SIL-TAL1阳性T-ALL患者年龄更低（*P*<0.001），HGB、WBC更高（*P*值分别为<0.001、0.009），在性别分布、PLT、染色体异常分布、免疫分型、完全缓解（CR）率方面，差异均无统计学意义（*P*值均>0.05）；接受造血干细胞移植后，SIL-TAL1阳性与SIL-TAL1阴性T-ALL患者3年OS率分别为60.9％和74.4％（*HR*＝2.070，*P*＝0.071）；3年无复发生存（RFS）率分别为49.2％和70.6％（*HR*＝2.275，*P*＝0.040），SIL-TAL1阳性T-ALL患者3年RFS率显著低于SIL-TAL1阴性T-ALL患者。

**结论:**

伴SIL-TAL1融合基因的T-ALL患者发病年龄更低，HGB、WBC更高，接受造血干细胞移植后预后较差，复发期短。

## Abstract (en)

**Objective:**

To assess the clinical characteristics and prognosis of patients with SIL-TAL1-positive T-cell acute lymphoblastic leukemia（T-ALL）.

**Methods:**

The clinical data of 19 SIL-TAL1-positive T-ALL patients admitted to the First Affiliated Hospital of Soochow University between January 2014 and February 2022 were retrospectively computed and contrasted with SIL-TAL1-negative T-ALL patients.

**Results:**

The median age of the 19 SIL-TAL1-positive T-ALL patients was 15（7 to 41 years）, including 16 males（84.2％）. SIL-TAL1-positive T-ALL patients had younger age, higher WBC, and hemoglobin compared with SIL-TAL1-negative T-ALL patients. There was no discrepancy in gender distribution, PLT, chromosome abnormality distribution, immunophenotyping, and complete remission（CR）rate. The 3-year overall survival（OS）was 60.9％ and 74.4％, respectively（*HR*＝2.070, *P*＝0.071）. The 3-year relapse-free survival（RFS）was 49.2％ and 70.6％, respectively（*HR*＝2.275, *P*＝0.040）. The 3-year RFS rate of SIL-TAL1-positive T-ALL patients was considerably lower than SIL-TAL1-negative T-ALL patients.

**Conclusion:**

SIL-TAL1-positive T-ALL patients were connected to younger age, higher WBC, higher HGB, and poor outcome.

急性T淋巴细胞白血病（T-ALL）是一种侵袭性的恶性血液病，占儿童ALL的10％～15％，成人ALL的25％[1]，预后差，复发率高。TAL1是T-ALL中染色体易位常见的靶点，编码Ⅱ类碱性-螺旋-环-螺旋（bHLH）转录因子，调控造血发育过程。TAL1基因位点90 kb的间质缺失与SIL的5′非编码区域结合，形成SIL-TAL1融合基因，从而导致TAL1蛋白的异常过表达[2]。SIL-TAL1融合基因是T-ALL中常见的染色体异常之一，发生率为16％～29％[3]。目前关于SIL-TAL1基因重排对T-ALL预后的影响存在争议。本研究通过回顾性分析我院收治的19例SIL-TAL1^+^ T-ALL患者临床资料并与同期收治的196例SIL-TAL1^−^ T-ALL患者比较，旨在了解SIL-TAL1融合基因对T-ALL临床特征及预后的影响，加深对此疾病的认识。

## 病例与方法

一、病例资料

回顾性分析2014年1月1日至2022年2月28日苏州大学第一附属医院收治的19例SIL-TAL1^+^ T-ALL患者病例资料。诊断采用细胞形态-免疫表型-细胞和分子遗传学特征（MICM）诊断模式，诊断分型采用WHO造血及淋巴组织肿瘤分类标准（2016版）[4]。同时回顾性分析同期本单位收治的196例SIL-TAL1^−^ T-ALL患者的临床资料。

二、方法

1. 免疫表型分析：采集患者骨髓标本，通过流式细胞术检测急性白血病免疫标志物。所检测抗原包括CD2、CD5、CD7、CD8、CD10、CD13、CD14、CD15、CD19、CD20、CD64、CD117、CD1a、TdT等。

2. 分子生物学检测：采用TRIzol法提取患者骨髓样本总RNA，以总RNA为模板，探针捕获目的基因片段建库，采用Nextseq 550测序系统进行脱氧核糖核酸检测，分析数据质量满足Q30。检测基因为《二代测序技术在血液肿瘤中的应用中国专家共识（2018版）》[5]推荐检测基因。应用Taqman探针实时荧光定量PCR进行融合基因的检测。

3. 染色体核型分析：采集患者骨髓标本，常规采用24 h短期培养法制备染色体标本，R显带进行核型分析。并按照《人类细胞遗传学国际命名体制（ISCN）2016》[6]对染色体异常核型进行描述。

4. 治疗：19例SIL-TAL1^+^ T-ALL患者及130例SIL-TAL1^−^ T-ALL患者诱导缓解化疗均采用VDCLP/VICLP方案（长春新碱+蒽环/蒽醌类药物+环磷酰胺+门冬酰胺酶+糖皮质激素），另有48例SIL-TAL1^−^患者诱导缓解化疗采用Hyper-CVAD方案：Hyper CVAD-A（环磷酰胺+长春新碱+表柔比星+地塞米松）、Hyper CVAD-B（甲氨蝶呤+阿糖胞苷）；诱导缓解化疗后第14、28天复查骨髓评估疗效，获得完全缓解（CR）者给予巩固治疗，巩固化疗方案包括VDP（长春新碱、柔红霉素、泼尼松）方案、阿糖胞苷、甲氨蝶呤、6-巯基嘌呤、门冬酰胺酶等；未获得CR或CR伴血细胞不完全恢复（CRi）的患者进入挽救治疗，挽救治疗方案包括FA（氟达拉滨+阿糖胞苷）、HD-MTX-A（大剂量甲氨蝶呤+阿糖胞苷）等。化疗后有移植条件者进行异基因造血干细胞移植（allo-HSCT）。

5. allo-HSCT：HLA配型全相合的患者预处理采用改良BUCY方案（阿糖胞苷+白消安+环磷酰胺+司莫司汀），HLA配型半相合者预处理采用改良BUCY+抗人T细胞兔免疫球蛋白（ATG-F）方案，伴中枢神经系统侵犯者采用全身照射（TBI）/CY方案预处理。预防移植物抗宿主病（GVHD）采用本科室常规方案：环孢素A+霉酚酸酯+小剂量甲氨蝶呤。

6. 随访：随访方式主要为病历查询和电话随访，随访截止时间为2022年4月26日，中位随访时间为48（1～99）个月。总生存（OS）期的计算以确诊日期为起始时间，任何原因导致死亡或末次随访时间为终止时间。无复发生存（RFS）期的计算以患者第1次获得CR（CR_1_）为起始时间，疾病复发或末次随访时间为终止时间。

三、统计学处理

采用SPSS 23软件进行统计学分析，两组非正态分布计量资料的对比采用秩和检验，以中位数（范围）表示；两组计数资料的对比采用*χ*^2^检验；生存资料的比较采用Log-rank检验，并采用Kaplan-Meier法进行生存曲线的绘制。以*P*<0.05为差异具有统计学意义。

## 结果

一、病例特征

19例SIL-TAL1^+^ T-ALL的患者中，男16例（84.2％），女3例（15.8％），初诊时，中位年龄15（7～41）岁，中位WBC 88.6（38.3～498.6）×10^9^/L，中位HGB 125（76～170）g/L，中位PLT 33（7～171）×10^9^/L。与196例SIL-TAL1^−^的T-ALL患者相比，SIL-TAL1^+^ T-ALL患者年龄更低（*P*<0.001），WBC、HGB更高（*P*值分别为<0.001、0.009），性别分布、PLT等方面差异均无统计学意义（*P*值均>0.05），具体见表1。

**表1:** SIL-TAL1融合基因阳性与阴性急性T淋巴细胞白血病（T-ALL）患者临床特征比较

临床特征	SIL-TAL1^+^ T-ALL（19例）	SIL-TAL1^−^ T-ALL（196例）	统计量	*P*值
年龄［岁，*M*（范围）］	15（7～41）	26.5（3～71）	4.033	0.000
性别［例（％）］			0.436	0.509
男	16（84.2）	146（74.5）		
女	3（15.8）	50（25.5）		
WBC［×10^9^，*M*（范围）］	88.6（38.3～498.6）	30.25（0.7～494.0）	−3.536	0.000
HGB［g/L，*M*（范围）］	125（76～170）	102（29～147）	−2.630	0.009
PLT［×10^9^，*M*（范围）］	33（7～171）	62（3～446）	1.748	0.081
骨髓原始细胞［％，*M*（范围）］	77（61～92）	80（21～99）	0.624	0.532
移植情况［例（％）］			0.378	0.539
是	16（84.2）	147（75.0）		
否	3（15.8）	49（25.0）		

二、实验室检查特征

1. 分子生物学特征：所有患者均表达异常T淋系标志。12例SIL-TAL1^+^ T-ALL患者通过二代测序检查血液系统肿瘤相关基因突变，共检出12种基因突变类型，常见突变依次为NOTCH1（50.0％）、FBXW7（41.7％）、PTEN（33.3％）、PDGFRB（16.7％），4例存在NOTCH1、FBXW共同突变。117例SIL-TAL1^−^患者通过二代测序共检出89种突变，常见突变依次为NOTCH1（62.4％）、JAK3（15.4％）、PHF6（15.4％）、NRAS（15.4％）（图1）。

**图1:**
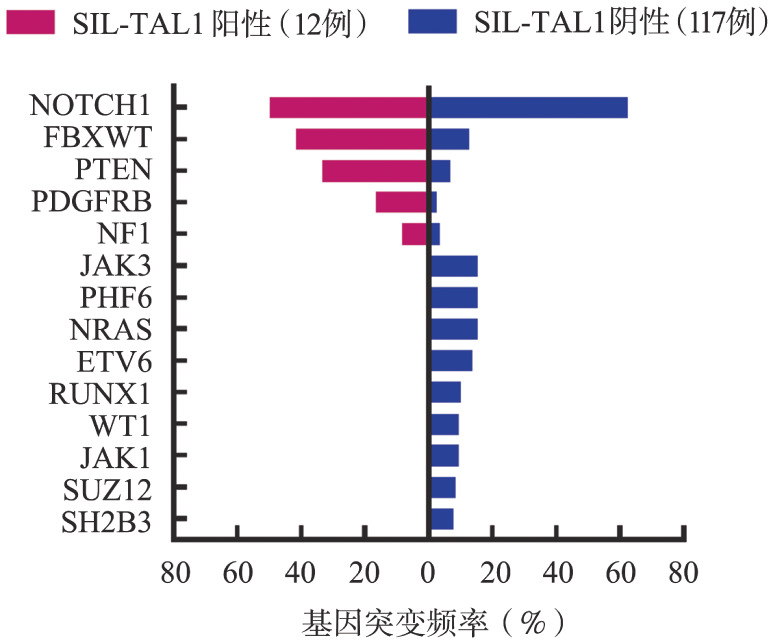
SIL-TAL1融合基因阳性与阴性急性T淋巴细胞白血病患者基因突变情况比较（％）

2. 细胞遗传学特征：19例SIL-TAL1^+^ T-ALL患者均进行了染色体核型分析，其中正常核型11例（57.8％），异常核型8例（42.2％）。具体异常核型见表2。167例SIL-TAL1^−^患者有染色体核型资料，正常核型111例（66.5％），异常核型56例（33.5％）。两组异常核型比例差异无统计学意义（*χ*^2^＝0.555，*P*＝0.456）。

**表2:** 19例SIL-TAL1融合基因阳性急性T淋巴细胞白血病患者临床信息

例号	性别	年龄（岁）	染色体	基因突变	CR情况	是否移植	移植方案	转归	RFS（月）	OS（月）
1	男	18	46,XY	NA	1个疗程CR	否	–	死亡	NA	NA
2	男	11	46,XY,inv(9)(p11q13)[20]	NA	1个疗程CR	是	半相合	死亡	24	23
3	女	10	46,XX,t(11;14)(p13;q11)[7]/46,XX[5]	NA	1个疗程CR	是	全相合	存活	93	94
4	男	7	46,XY	NA	1个疗程CR	是	半相合	死亡	18	46
5	女	41	46,XX	NA	1个疗程CR	否	–	死亡	NA	NA
6	男	25	46,XY	NOTCH1	1个疗程CR	是	全相合	存活	76	77
7	男	17	46,Y,t(X;8)(q13;q24)[2]/46,XY[5]	NA	1个疗程CR	是	半相合	存活	73	74
8	男	15	46,XY	NOTCH1、FBXW7	1个疗程CR	是	半相合	死亡	NA	NA
9	男	20	46,XY	PTEN	1个疗程CR	是	半相合	死亡	NA	NA
10	男	11	46,XY	NA	1个疗程CR	是	半相合	存活	67	68
11	男	15	47,XY,+c[2]/46,XY,der(18) t(1;18)(q23;q23)[2]/46,XY[6]	FBXW7、PDGFRB	NR	是	半相合	死亡	NA	NA
12	男	14	46,XY,der(4),inv(8)(p23q23),der(11)t(11;14)(p15;q21), der(14)add(14)(p11)t(11;14)[8]/46,XY[2]	PTEN	1个疗程CR	是	半相合	死亡	1	17
13	男	32	46,XX	NA	1个疗程CR	是	全相合	死亡	17	18
14	男	14	46,XY	FBXW7、NOTCH1、PIK3CA、STAG2	1个疗程CR	是	半相合	存活	39	40
15	女	15	47,XX,+mar[10]	FBXW7、NOTCH1	1个疗程CR	是	半相合	存活	3	35
16	男	15	46,XY,6q−	NOTCH1、PDGFRB	1个疗程CR	是	半相合	死亡	9	13
17	男	29	46,XY	PTEN	2个疗程CR	是	半相合	死亡	1	21
18	男	15	46,XY	NOTCH1、FBXW7、CIITA、NOTCH2	3个疗程CR	否	–	存活	9	12
19	男	11	46,XY,add(5)(q33)[10]	NF1、ATG2B、KMT2D、NT5C2、PTEN	1个疗程CR	是	全相合	存活	9	10

**注** NA：未检测；CR：完全缓解；NR：未缓解；RFS：无复发生存期；OS：总生存期；−：不适用

三、治疗疗效

19例SIL-TAL1^+^T-ALL患者均接受了规范化治疗，4周诱导化疗后CR率为89.5％（16/19）；196例SIL-TAL1^−^ T-ALL患者中178例接受了规范化治疗，其中158例可评价缓解情况，4周诱导化疗后CR率为70.3％（111/158）。两组患者4周诱导化疗CR率差异无统计学意义（*χ*^2^＝1.630，*P*＝0.202）。经4周诱导化疗未缓解（NR）者，大多数经2～4个疗程化疗后达CR，SIL-TAL1阳性和阴性患者总的CR率分别为89.5％和89.3％，差异无统计学意义（*χ*^2^＝0.000，*P*＝1.000）。

四、移植情况

16例SIL-TAL1^+^患者进行了allo-HSCT，包括12例HLA半相合移植，4例HLA全相合移植。其中15例达CR者经1～3个疗程巩固化疗后进行allo-HSCT，1例NR者经3个疗程诱导化疗后进行挽救性allo-HSCT。SIL-TAL1^+^与SIL-TAL1^−^ T-ALL患者移植率分别为84.2％和75.0％，差异无统计学意义（*χ*^2^＝0.378，*P*＝0.539）。

五、生存情况

接受allo-HSCT的16例SIL-TAL1^+^ T-ALL与147例SIL-TAL1^−^ T-ALL患者生存数据进行比较，3年OS率分别为60.9％和74.4％（*HR*＝2.070，*P*＝0.071）（图2A）；3年RFS率分别为49.2％和70.6％（*HR*＝2.275，*P*＝0.040）（图2B），SIL-TAL1^+^ T-ALL患者3年RFS率显著低于SIL-TAL1^−^T-ALL患者。3例未接受allo-HSCT的SIL-TAL1^+^ T-ALL患者，2例失访，1例目前仍为CR状态，OS期为12个月。

**图2:**
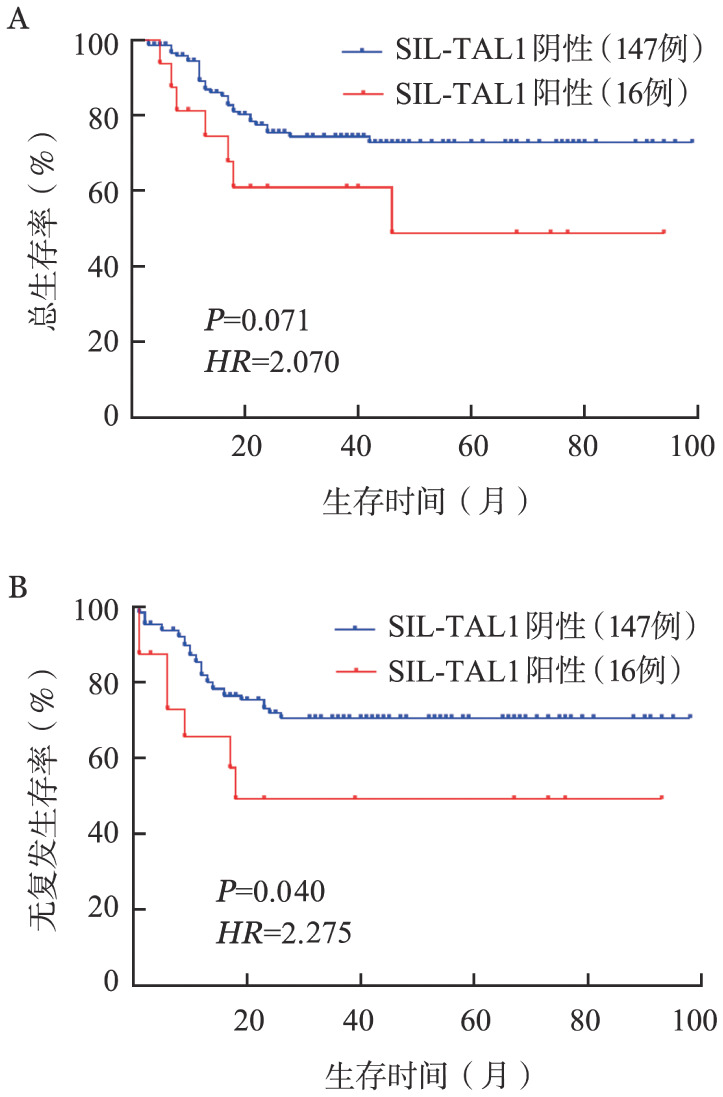
接受异基因造血干细胞移植的SIL-TAL1^+^ T-ALL患者与SIL-TAL1^−^ T-ALL患者总生存（A）和无复发生存（B）曲线

## 讨论

染色体1p32上的TAL1位点的破坏是T-ALL中最常见的遗传异常之一，在1％～3％的儿童T-ALL中，TAL1中断与t（1；14）（p33；q11）相关[7]；在16％～29％的T-ALL中，TAL1的5′端中断与SIL的5′端结合，形成SIL-TAL1融合基因[8]。两种TAL1位点的破坏都能引起TAL1蛋白的异常编码，导致其在T细胞中异位表达[9]。迄今为止，仅有少数临床研究描述SIL-TAL1^+^ T-ALL的临床特征。本研究结果表明，SIL-TAL1融合基因在T-ALL中的发生率为8.8％，低于文献报道的发生率，可能与SIL-TAL1^+^ T-ALL患者多见于儿童，但本中心收治患者多为成人有关。SIL-TAL1^+^与SIL-TAL1^−^ T-ALL患者相比，具有年龄小、高WBC、高HGB的特点，在性别分布、PLT、CR率方面，差异无统计学意义。此研究结果与已有文献报道结果一致[10]。

本研究比较SIL-TAL1阳性T-ALL患者与阴性T-ALL患者染色体异常比例分布，两者差异无统计学意义；通过二代测序分析，尽管SIL-TAL1^+^ T-ALL患者例数较少，但仍能发现NOTCH1、FBXW7、PTEN突变频率较高，其中有21.1％（4/19）存在NOTCH1、FBXW7共同突变。有文献报道：伴有NOTCH1、FBXW7共同突变的T-ALL患者预后较好[11]，与本文结论相悖，可能的原因为病例数较少、SIL-TAL1^+^ T-ALL患者年龄较小，以及本研究纳入的患者80％以上进行了allo-HSCT。

STL-TAL1融合基因对T-ALL患者预后的影响仍具有争议，在一些报道中认为SIL-TAL1阳性T-ALL患者预后更好[12]，而Mansur等[13]认为SIL-TAL1对巴西儿童T-ALL的OS期具有不良影响，Ballerini等[14]认为SIL-TAL1对T-ALL的OS无影响。Wang等[2]分析16例未进行造血干细胞移植的SIL-TAL1^+^ T-ALL患者，中位OS期显著低于未移植的SIL-TAL1^−^ T-ALL患者（4个月对25个月，*P*<0.05），提示SIL-TAL1^+^ T-ALL患者预后极差。本研究比较了接受造血干细胞移植的16例SIL-TAL1^+^ T-ALL与147例SIL-TAL1^−^ T-ALL患者生存数据，两组3年OS率分别为60.9％和74.4％，差异无统计学意义（*HR*＝2.070，*P*＝0.071），推测可能的原因为病例数量较少，且均进行了造血干细胞移植，以上因素综合作用可能会影响SIL-TAL1融合基因在T-ALL预后中的作用。通过比较造血干细胞移植后SIL-TAL1^+^ T-ALL患者和SIL-TAL1^−^ T-ALL患者的RFS时间，发现SIL-TAL1^+^ T-ALL患者更早复发，3年RFS率显著低于SIL-TAL1^−^ T-ALL患者。由于未接受造血干细胞移植的SIL-TAL1^+^ T-ALL患者可随访病例数较少，故未与融合基因阴性患者进行比较。由于本研究纳入阳性患者例数较少，对于SIL-TAL1^+^ T-ALL患者和SIL-TAL1^−^ T-ALL患者的OS及RFS的比较，有待扩大样本量进一步深入研究。

综上所述，SIL-TAL1^+^ T-ALL患者具有发病年龄小、高白细胞计数、高血红蛋白浓度的特点，SIL-TAL1融合基因阳性T-ALL患者接受造血干细胞移植后预后较差，复发期短。
